# Comparative analysis of macrophage transcriptome of four mice strains after *L. Major* infection

**Published:** 2011-01-10

**Authors:** Fouad Benhnini, RM Sghaier, FZ Guerfali, D Laouini, PA Cazenave, K Dellagi

**Affiliations:** 1 Laboratoire d’Immuno-Pathologie, Vaccinologie et Génétique Moléculaire, Institut Pasteur de Tunis, Tunisia; 2 Universite Pierre et Marie Curie & Institut Pasteur, 75724 Paris Cedex 15, France

## 

Leishmaniasis is a parasitic disease caused by a protozoan parasite of the genus *Leishmania* (*L*.), using the macrophage as the main host cell where it can survive and replicate. The infection outcome depends on the balance between the ability of the host to activate the macrophage microbocidal mechanisms to kill the parasite, and the pathogen’s ability to suppress the host immune response and survive in the harsh environment of phagolysosomes. This ability to circumvent the host immune response may be the result of pressure exerted by the parasite on macrophage gene expression in a way that promotes their survival and multiplication.

In this study, we compared the expression kinetics of 82 Bone-Marrow-Derived Macropahge (BMDM) gene transcripts, from susceptible (BALB/c and PWK) and resistant (C57BL/6 and MBT) mice strains, following *in vitro* infection with *L. major* parasites, the causative agent of human zoonotic cutaneous leishmaniasis. These genes belong to several functional families e.g., IFN? pathway, TLRs, chemokines, nitric oxid production pathway or involved in various metabolic pathways.

Our results showed a clear contrast between the different profiles according to the infection stage (early vs. middle or late) in the four mice strains: regardless to targeted transcripts, the BMDM gene expression profile reflects, at early stages (3h post-infection), the genetic mice background and their susceptibility to *Leishmania* infection; whereas there is no such correlation at later stages.

Indeed, and independently of the gene function, macrophages of susceptible BALB/c and PWK mice showed a general inhibited expression profile while the resistant C57BL/6 and MBT mice expression profile levels are higher compared to non-infected BMDM.

Strikingly, this observation was not seen at later times of infection (24h and 72h post-infection) and the expression level depends on each transcript; a direct relationship between the mouse background and the level of expression of a given gene being less obvious. Whether, this early strain-specific effect of parasites on BMDM is due to a general phenomenon that may be encountered with any phagocytosed particle or specific to *L. major* will be discussed. This study provides a strong argument that susceptibility *versus* resistance to *Leishmania* infection of mice with different genetic backgrounds, would be partly due to the innate immunity.

These results indicate that these differences in activation of innate immunity illustrate, at least in part, the differences in clinical expression of experimental leishmaniasis in the four mice strains.

